# Age and Attitudes Towards an Internet-Mediated, Pedometer-Based Physical Activity Intervention for Chronic Obstructive Pulmonary Disease: Secondary Analysis

**DOI:** 10.2196/19527

**Published:** 2020-09-09

**Authors:** Stephanie A Robinson, Emily S Wan, Stephanie L Shimada, Caroline R Richardson, Marilyn L Moy

**Affiliations:** 1 Center for Healthcare Organization and Implementation Research Edith Nourse Rogers Memorial Veterans Hospital Bedford, MA United States; 2 Boston University School of Medicine Boston, MA United States; 3 Pulmonary and Critical Care Medicine Section Veterans Affairs Boston Healthcare System Boston, MA United States; 4 Channing Division of Network Medicine Brigham & Women's Hospital Boston, MA United States; 5 Harvard Medical School Boston, MA United States; 6 Department of Health Law, Policy, and Management Boston University School of Public Health Boston, MA United States; 7 Department of Family Medicine University of Michigan Ann Arbor, MI United States

**Keywords:** aging, COPD, chronic conditions, physical activity, eHealth

## Abstract

**Background:**

Chronic obstructive pulmonary disease (COPD) is prevalent among older adults. Promoting physical activity and increasing exercise capacity are recommended for all individuals with COPD. Pulmonary rehabilitation is the standard of care to improve exercise capacity, although there are barriers that hinder accessibility. Technology has the potential to overcome some of these barriers, but it is unclear how aging adults with a chronic disease like COPD perceive technology-based platforms to support their disease self-management.

**Objective:**

Guided by the unified theory of acceptance and use of technology, the current retrospective secondary analysis explores if age moderates multiple factors that influence an individual with COPD’s openness toward an internet-mediated, pedometer-based physical activity intervention.

**Methods:**

As part of an efficacy study, participants with COPD (N=59) were randomly assigned to use an internet-mediated, pedometer-based physical activity intervention for 12 weeks. At completion, they were asked about their experience with the intervention using a survey, including their performance expectancy and effort expectancy, facilitating conditions (ie, internet use frequency and ability), and use of the intervention technology. Logistic regression and general linear modeling examined the associations between age and these factors.

**Results:**

Participants ranged in age from 49 to 89 years (mean 68.66, SD 8.93). Disease severity was measured by forced expiratory volume in the first second percent predicted (mean 60.01, SD 20.86). Nearly all participants (54/59) believed the intervention was useful. Regarding effort expectancy, increasing age was associated with reporting that it was easy to find the time to engage in the intervention. Regarding facilitating conditions, approximately half of the participants believed the automated step count goals were too high (23/59) and many did not feel comfortable reaching their goals (22/59). The probability of these perceptions increased with age, even after accounting for disease severity. Age was not associated with other facilitating conditions or use of the technology.

**Conclusions:**

Age does not influence performance expectancy or use of technology with an internet-mediated, pedometer-based physical activity intervention. Age is associated with certain expectations of effort and facilitating conditions. Consideration of age of the user is needed when personalizing step count goals and time needed to log in to the website.

**Trial Registration:**

ClinicalTrials.gov NCT01772082; https://clinicaltrials.gov/ct2/show/NCT01772082

## Introduction

### Chronic Obstructive Pulmonary Disease and eHealth

Chronic obstructive pulmonary disease (COPD), prevalent mainly in older adults, is the fourth leading cause of death in the United States [1]. Independent of lung function, physical inactivity in COPD is associated with poor outcomes, worse health-related quality of life [2,3], higher health care use [4], and mortality [5]. Physical activity is recommended for all individuals with COPD [6,7]. Pulmonary rehabilitation is the standard of care to increase physical activity and exercise capacity; however, there are barriers to conventional pulmonary rehabilitation, including distance and time required to travel to medical center–based in-person sessions.

Technology or eHealth can potentially be used to overcome many barriers to accessibility. Technology-based interventions may support self-management behaviors, such as engagement in physical activity [8]. Randomized controlled trials (RCTs) have demonstrated the efficacy of these interventions in persons with COPD [3,9-12]. However, implementation into usual care has been slow [13]. One of the factors impeding implementation might be the concern that increasing age may be associated with lower technology knowledge and use. Understanding what influences one’s acceptance and use of technology can help select effective implementation strategies [14].

Among patients with chronic diseases, such as COPD, it is unclear if age influences attitudes toward web-based platforms to support disease self-management. Some literature suggests that increasing age is negatively associated with effort expectancy and use of technology [15-17], but other studies suggest that age does not impact technology acceptance [18]. Additionally, there is a perceived decreased rate of technology ownership among older individuals, although reviews have found that a significant portion of older adults already use technology and that rates of ownership are increasing every year across all age groups [18,19]. Finally, age-related visual, motor, or cognitive limitations may make it difficult for an older adult to efficiently use some technologies, which subsequently discourages interest and use [18]. In order to provide an optimal context in which eHealth interventions can effectively improve self-management behaviors and health outcomes, it is important to understand the potential factors that influence attitudes towards technology acceptance and use among aging patients in this population [18]. Careful consideration of how age influences these factors can be used to develop or adapt the technology to better meet the needs of patients with COPD.

### Theoretical Background

**Figure 1:**
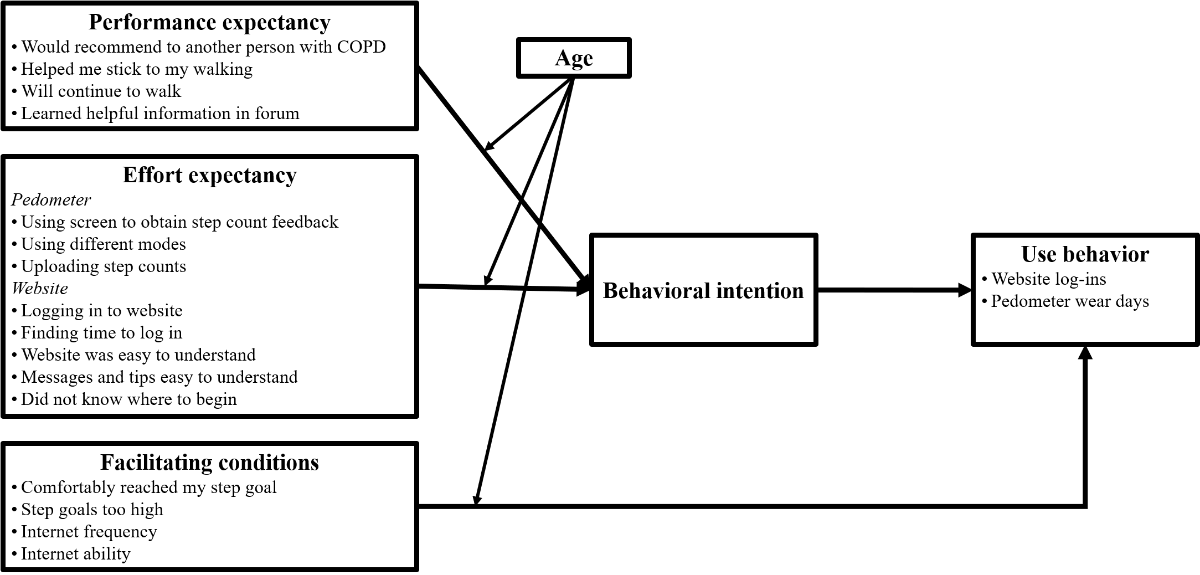
Conceptual framework of the unified theory of acceptance and use of technology, modified to represent the current study and the corresponding items assessed. COPD: chronic obstructive pulmonary disease.

The unified theory of acceptance and use of technology (UTAUT) provides a conceptual framework for predicting (1) behavioral intention to use a technology and (2) technology use behavior [20,21]. These factors include performance expectancy, effort expectancy, facilitating conditions, and social influence. Performance expectancy refers to the degree to which an individual believes that using the system will help him or her. Effort expectancy refers to the degree of ease associated with using the technology. Facilitating conditions refer to conditions that may support acceptance and use of the technology. Social influence refers to what degree an individual feels that others believe he or she should use the new technology [20]; due to the retrospective nature and data limitations of the current study, we were unable to examine social influence. Figure 1 shows the UTAUT constructs we were able to assess in the study. The UTAUT posits that facilitating conditions have a direct effect on use behavior. Internet efficacy, or digital literacy, has been identified as a facilitating factor predicting the use of eHealth [22]. Early UTAUT research focused primarily on predicting behavioral intention to use a technology and actual technology use, primarily in organizational contexts. Since then, Venkatesh et al [20] encouraged extensions in new contexts, user populations, and cultural settings. The current study continues this extension by examining technology acceptance from the individual’s perspective in a Veteran COPD population.

### Current Study

Using the UTAUT, the current secondary analysis explored whether age moderated factors associated with behavioral intention to use technology by persons with COPD who used Every Step Counts (ESC). ESC is an internet-mediated, pedometer-based physical activity intervention that we have previously shown to be efficacious in Veterans with COPD [12]. We hypothesized that increasing age would be associated with decreased performance expectations, increased effort expectations, decreased facilitating conditions, and decreased use (website log-ins and pedometer wear days) of a technology-based physical activity intervention for participants with COPD.

## Methods

### Design

This secondary analysis used data from a previously reported RCT registered on ClinicalTrials.gov (NCT01772082) [12]. The RCT was approved by the Veterans Affairs Boston Healthcare System Institutional Review Board (Protocol No. 2328). All participants gave written informed consent. Participants with COPD, defined as a ratio of forced expiratory volume in the first second to forced vital capacity (FEV_1_/FVC) less than 0.7 or emphysema on clinical chest computed tomography, were randomly assigned to use either a website coupled with a pedometer (ESC intervention) or a pedometer alone. The sample of the current analysis includes only the participants who were randomly assigned to use the ESC intervention (n=59).

### Intervention

ESC is a multicomponent, internet-mediated, pedometer-based physical activity intervention. ESC was developed based on the theory of self-regulation, which emphasizes an iterative approach to behavior change using personalized goals, iterative step count feedback for self-monitoring, educational tips, and motivational messages to enhance disease self-management and self-efficacy, as well as an online community forum for social support. Participants were asked to use ESC for 12 weeks and to wear a pedometer (Omron HJ-720 ITC; Omron Healthcare Inc). Participants were asked to wear the pedometer daily, clipped to their clothing around their waist. Use of the pedometer also required participants to ensure the pedometer had sufficient battery power. Participants could view their steps on the face of the pedometer and change the pedometer display (steps, aerobic steps, calories, miles) by pressing buttons. Additionally, participants had to navigate the website to upload their step count data from their pedometer, view their feedback graphs and weekly step count goals, and access the educational tips, motivational messages, and online forum. After the 12-week intervention, as part of their visit, they were asked for feedback using a survey about their experience using the pedometer and website.

### Measures

Age, measured continuously and assessed at study entry, was included as an independent variable in the analyses. Marital status, race, and disease severity were included as covariates in all analyses. Marital status was measured categorically: single/never married, separated, divorced/annulled, widowed, or married. FEV_1_ percent predicted (FEV_1_% predicted), a spirometric measurement of airway obstruction, was used to characterize disease severity [23,24].

#### Performance Expectancy

Participants were asked about how useful they found the intervention to be for increasing their physical activity. They were asked whether they agreed (true or false) with the following statements: (1) “I would recommend the Every Step Counts for Lung Health walking program to another person with COPD”; (2) “The Every Step Counts for Lung Health program helped me stick to my walking for exercise”; (3) “I will continue to walk for exercise after the research study ends”; and (4) “I learned helpful information when I used the online community forum.” Responses to the statements were examined individually.

#### Effort Expectancy

Participants were asked about their experience using the technology (pedometer and website). Regarding the pedometer, participants were asked whether they agreed (yes or no) that during a typical week during the research study, they regularly (4-7 days/week) had any of the following problems using the Omron pedometer: (1) “difficulty using the Omron screen to obtain step-count feedback”; (2) “difficulty using the different Omron ‘modes’ (steps, aerobic steps, kcal, miles)”; and (3) “technical difficulty uploading step-count data from the Omron pedometer to my computer.”

Regarding the website, participants were asked whether they agreed (yes or no) that they regularly (defined as more than half of the times they accessed the site) had any of the following experiences using aspects of the website: (1) “Problems with logging in to the website”; (2) “It was easy for me to find the time to log in to the website once a week”; (3) “The Every Step Counts for Lung Health website was easy to understand”; (4) “The motivational messages and educational tips were easy to understand”; and (5) “After I logged in to the website, I did not know where to begin to use it.”

#### Facilitating Conditions

Participants self-reported facilitating conditions that could influence their use and self-efficacy using the intervention. Regarding the website-generated, personalized, and iterative step goals, participants were asked whether they agreed (yes or no) with the following statements: (1) “I was able to comfortably reach my step-count goal each week as directed by *Every Step Counts*” and (2) “The daily step-count goals were too high for me to walk each day.” Other facilitating conditions included internet use and competence; these were measured ordinally. Participants were asked how often they use the internet on a scale from 1 (never) to 4 (every day) and how they would rate their ability to use the internet on a scale from 1 (no ability) to 5 (expert ability).

#### Use Behavior

Use behavior, or use of the technology-based components of the intervention, was measured continuously. The number of days participants wore the pedometer over the study period was used to measure use of the pedometer. The number of times the participants logged on to the website over the course of the study was used to measure use of the website.

### Statistical Analyses

We used descriptive statistics to examine the frequency of responses to the different UTAUT constructs. We used logistic regression to estimate the odds ratio (OR) to measure the association between age and dichotomous variables that examined performance expectancy and effort expectancy. ORs were considered significant if the 95% confidence interval did not include 1.00 and the *P* value was less than .05. General linear modeling was used to examine the association between age on the continuous and ordinal outcome variables that measured facilitating conditions and use behavior (ie, pedometer wear days and website log-ons). General linear model estimates were considered significant if the 95% confidence interval did not include 0.00 and the *P* value was less than .05. Marital status, race, and FEV_1_% predicted were included as covariates in all models. All analyses were performed using SAS 9.4 (SAS Institute).

## Results

### Participant Characteristics

Table 1 displays participant characteristics. Participants ranged in age from 49 to 89 years (mean 68.66, SD 8.93). Most of the participants were male (58/59, 98%), White (55/59, 93%), married (26/59, 44%), and retired (35/59, 59%), and most earned an annual income of at least US $30,000 per year (38/59, 65%). There was a statistically significant difference in age between reported employment categories (*χ*^2(3)^=8.7; *P*=.03). The mean age of retired participants was 70.1 years (SD 7.9) compared with 61.8 years (SD 6.1) who indicated they were working full time.

**Table 1:** Characteristics of study participants (N=59).

Characteristics			Statistics
**Demographic characteristics**			
	Age (years), mean (SD)		68.66 (8.93)
	**Gender, n (%)**		
		Male	58 (98)
		Female	1 (2)
	**Race, n (%)**		
		White	55 (93)
		African American	3 (5)
		American Indian or Alaska Native	1 (2)
	**Income (US), n (%)**		
		<$15,000	10 (17)
		$15,000-$29,999	11 (19)
		$30,000-$49,999	18 (31)
		$50,000 or more	20 (34)
	**Marital status, n (%)**		
		Single/never married	6 (10)
		Separated	1 (2)
		Divorced or annulled	20 (34)
		Widowed	6 (10)
		Married	26 (44)
	**Employment, n (%)**		
		Full-time job	5 (8)
		Part-time job	11 (19)
		Not working due to disability	8 (14)
		Retired	35 (59)
**Medical characteristics**			
	FEV_1_% predicted^a^, mean (SD)		60.01 (20.86)
	Oxygen, n (%)		4 (7)
	**Comorbidities, n (%)**		
		Coronary artery disease	10 (17)
		Diabetes	17 (29)
		Arthritis	18 (31)
	Previous pulmonary rehabilitation, n (%)		6 (10)
**Intervention characteristics**			
	Change in daily step counts, mean (SD)		440.17 (1819.74)

^a^FEV_1_% predicted: forced expiratory volume in the first second percent predicted.

### Performance Expectancy

The majority of participants randomized to ESC believed the intervention to be useful; 95% (56/59) of the sample reported that they would recommend the intervention to other Veterans with COPD. Only 32% (19/59) found the online forum to contain helpful information, and 63% (37/59) reported that they did not use the online forum. Approximately three-quarters (45/59, 76%) believed that the intervention helped them stick to their walking, and 98% (58/59) reported that they will continue to walk. Contrary to our hypothesis, the OR of these responses did not vary by age, adjusting for marital status, race, and FEV_1_% predicted (Table 2).

**Table 2:** Summary of age associations on UTAUT constructs.

UTAUT^a^ construct			Response	AOR^b^ (95% CI)	B (95% CI)	*P* value
**Performance expectancy, n (%)**						
	Would recommend to another person with COPD^c^		56 (95)	0.99 (0.798 to 1.235)	N/A^d^	.95
	Helped me stick to my walking		45 (76)	0.95 (0.869 to 1.030)	N/A	.20
	Will continue to walk		53 (90)	0.92 (0.801 to 1.057)	N/A	.24
	Learned helpful information in forum		19 (32)	0.99 (0.924 to 1.070)	N/A	.88
**Effort expectancy, n (%)**						
	**Pedometer**					
		Using screen to obtain step count feedback	12 (20)	1.08 (0.971 to 1.200)	N/A	.16
		Using different modes	6 (10)	1.12 (0.976 to 1.285)	N/A	.11
		Uploading step counts	21 (36)	1.05 (0.971 to 1.133)	N/A	.22
	**Website**					
		Logging in to website	9 (16)	1.04 (0.941 to 1.142)	N/A	.47
		Finding time to log in	48 (81)	1.17 (1.030 to 1.332)	N/A	.02
		Website was easy to understand	54 (92)	1.01 (0.874 to 1.156)	N/A	.94
		Messages and tips easy to understand	34 (58)	1.02 (0.951 to 1.094)	N/A	.57
		Did not know where to begin	7 (12)	0.99 (0.880 to 1.121)	N/A	.91
**Facilitating conditions, n (%)**						
	Comfortably reached my step goal		22 (37)	0.91 (0.827 to 0.990)	N/A	.03
	Step goals too high		23 (40)	1.08 (1.000 to 1.172)	N/A	.049
	**Internet frequency**			N/A	0.00 (0.656 to 4.897)	.90
		Never	3 (5)			
		≤4 times per month	8 (16)			
		Several times a week	12 (20)			
		Every day	36 (61.0)			
	**Internet ability**			N/A	–0.03 (–0.063 to 0.010)	.15
		None	3 (5)			
		Basic	27 (46)			
		Moderate	17 (29)			
		Advanced	8 (14)			
		Expert	4 (7)			
**Use behavior, mean (SD)**						
	Pedometer wear days		77.2 (18.8)	N/A	0.31 (–0.345 to 0.971)	.34
	Weekly number of log-ons		5.4 (3.1)	N/A	0.02 (–0.341 to 0.384)	.91

^a^UTAUT: unified theory of acceptance and use of technology.

^b^AOR: adjusted odds ratio.

^c^COPD: chronic obstructive pulmonary disease.

^d^N/A: not applicable.

### Effort Expectancy

**Figure 2:**
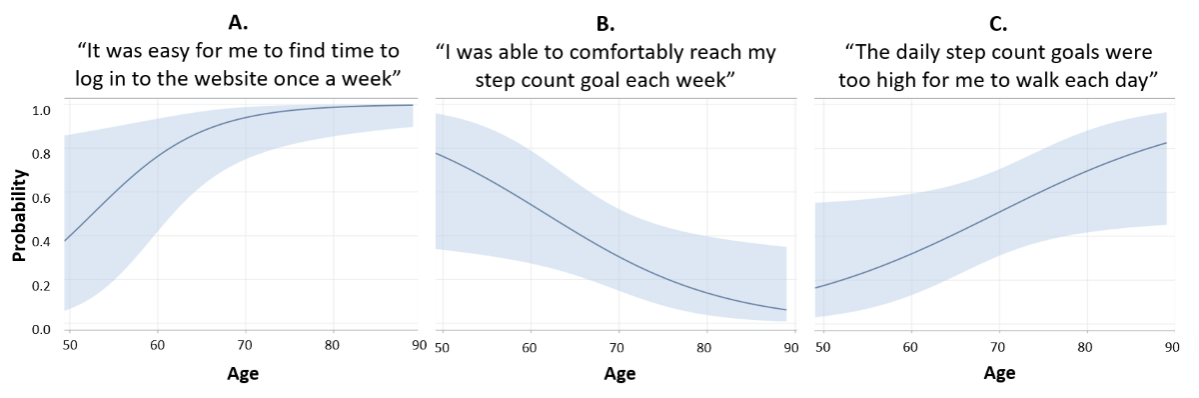
Predicted probability (logistic regression) of agreeing that (A) “It was easy for me to find time to log in to the website once a week”; (B) “I was able to comfortably reach my step count goal each week”; and (C) “The daily step goals were too high.” Figures include 95% confidence limits. Fit computed controlling for marital status, race, and forced expiratory volume in the first second percent predicted.

We hypothesized that higher age would be associated with increased effort expectancy. When we examined participants’ perceived effort expectancy of the pedometer, 20% (12/59) thought it was difficult to obtain step count feedback from the pedometer, 10% (6/59) found it difficult to use the different modes on the pedometer, and 36% (21/59) had difficulty uploading their step count data to the website. None of these were associated with age (Table 2). When asked if it was easy to find time to use the website, 81% (48/59) agreed. Age was significantly associated with increased odds of agreeing that it was easy to find time to use the website (OR 1.17, 95% CI 1.030-1.332; *P=*.02). As shown in Figure 2, agreement that it was easy to find time to use the website began to level off around an age of 70 years. The majority of participants (54/59, 92%) reported that the website was easy to understand. More than half (34/59, 58%) believed the motivational messages and educational tips were easy to understand; 41% (24/59) did not view these messages and tips.

### Facilitating Conditions

A total of 37% (22/59) of participants agreed that they were able to comfortably reach their step count goal. Increasing age was significantly associated with decreased odds of agreeing that they felt comfortable reaching their step goal (OR 0.91, 95% CI 0.827-0.990; *P*=.03; Figure 2). Similarly, 39% (23/59) of participants agreed that their daily step count goals were too high. Increasing age was significantly associated with increased odds of agreement (OR 1.08, 95% CI 1.003-1.172; *P=*.049; Figure 2). In addition, 15% (9/59) reported trouble logging in and 12% (7/59) reported not knowing where to begin on the website once logged-in. At study entry, the majority of those randomized to the ESC intervention self-reported using the internet every day (36/59, 61%). Most participants self-reported basic (27/59, 46%) or moderate (17/59, 29%) internet abilities. Age did not significantly predict self-reported frequency of internet use or perceived internet ability (Table 2).

### Use Behavior

Across the 12-week, or 84-day, study period, participants wore the pedometer an average of 77.19 days (SD 18.78). Participants were asked to log on at least once a week to upload their step count data to the study website. The average number of website log-ons throughout the 12-week intervention period was 15.69 (SD 9.63). Contrary to our hypothesis, pedometer wear days and number of log-ons did not vary by age (Table 2).

Guided by the UTAUT, we examined the direct relationship between facilitating conditions and use behavior. Feeling comfortable reaching the step goals was not significantly associated with website log-ons (B=0.81, 95% CI –1.25 to 2.88; *P*=.43). Similarly, perceiving the step goals as too high was also not associated with website log-ons (B=–0.51, 95% CI –2.58 to 1.56; *P*=.63). Self-reported frequency of internet use at baseline was marginally associated with number of website log-ons during the study (B=1.10, 95% CI –0.02 to 2.22; *P*=.05). Self-reported internet ability was not significantly associated with the number of log-ons (B=0.77, 95% CI –0.15 to 1.69, SE 0.45; *P*=.10).

Feeling comfortable reaching their step goals was not significantly associated with website pedometer wear days (B=2.08, 95% CI –8.81 to 12.97, SE 5.41; *P*=.70). Similarly, perceiving the step goals as too high was also not associated with pedometer wear days (B=–0.10, 95% CI –10.62 to 10.43, SE 5.23; *P*=.99). Self-reported frequency of internet use at baseline did not significantly predict valid pedometer wear days during the study (B=–2.18, 95% CI –9.02 to 3.65; *P*=.46), nor did self-reported internet ability (B=–1.55, 95% CI –6.43 to 3.33; *P*=.53).

## Discussion

### Overview

The current study examined COPD patients’ acceptance and use of an internet-mediated, pedometer-based physical activity intervention, ESC, using the UTAUT as a conceptual model. Results support that participants with COPD are generally able to use the technologies involved with ESC (ie, pedometer and website). An overwhelming majority of the sample reported that they would recommend the intervention to other Veterans with COPD, believed that the intervention helped them walk more, that they would continue to walk, and that the website was easy to understand. This was true for participants across all ages. There were themes from the UTAUT, such as perceiving goals as unattainable, that differed by age. This provides important information on how to improve and implement such a technology-mediated intervention in COPD.

### Age Was Associated With Perceived Difficulty in Meeting Step Count Goals

Approximately 63% (37/59) of the participants did not feel they could comfortably reach their given step count goal and nearly 40% (23/59) believed that their step count goals were too high. Both perceptions increased as age increased, independent of disease severity as reflected by lung function. ESC used an algorithm that considered the participant’s steps walked from the previous week to automatically calculate and increment their goal each week. Goals were set at the minimum value of 3 possible numbers: (1) the previous goal + 400 steps, (2) the average of the most recently uploaded 7 days of step counts + 400 steps, or (3) 10,000 steps [12]. Previous pilot work found that a weekly increase of 400 steps was an attainable goal for a COPD cohort [11]. Despite this personalization, older participants were still more likely to believe their goals were too high and felt uncomfortable reaching their step count goals. This was true over and above the severity of the participant’s disease, as measured by FEV_1_% predicted.

Goal setting has been a prominent intervention strategy to increase motivation and effect behavior change [25]. As done with ESC, technology can be used to set automated exercise goals in interventions that are personalized to a COPD population. Although ESC attempted to set realistic, small, and gradual goals that would be effective for long-term engagement compared with larger goals, participant feedback shows that goals need to be further tailored based on the user’s age. Goal setting is strongly tied to one’s self-efficacy, or confidence that they can accomplish a goal, which is directly related to one’s behavior [26,27]. Setting goals that the participants perceive as unrealistic and too difficult can be detrimental to their self-efficacy [26]. Our results suggest that automated goals may need to account for age to increase effort expectancy of the intervention. Future studies could examine whether smaller weekly increments would help participants perceive the goals as being more attainable as age increases.

### Increasing Age Was Associated With More Time to Engage With the Website

Increased age was associated with endorsing that it was easier to find the time to use the website. Our older participants were retired, whereas the younger, middle-aged participants were still of working age. This perceived lack of time closer to midlife is common [28], particularly for those who feel a conflict between the many goals or tasks one must achieve throughout the day (eg, finishing work, picking up children, etc). It is not surprising that as age increases, participants, who are more likely to be retired, are more likely to agree that it was easy to find the time to engage in the website. Indeed, the majority of our sample did indicate that they were retired.

Time constraints for busy adults are likely to be an ongoing obstacle to engaging in physical activity. This is an important consideration when designing alternative interventions to address barriers to activity promotion strategies. One of the barriers to conventional pulmonary rehabilitation is the time commitment, both the amount of time required to travel to attend in-person sessions and the amount of time required to complete the rehabilitation sessions [29,30]. Presumably, time is less of a burden for a web-based intervention that does not require travel or in-person sessions, although we still found that age was positively associated with believing it was easy to find time to use the website. Future work would benefit from examining and comparing participant perspectives, particularly of middle-aged participants, on the time burdens of both traditional in-person rehabilitation and technology-based interventions.

### Age Was Not Associated With Use Behavior

Age was not associated with use of the technology-based intervention (pedometer wear days or website log-ons). Similarly, self-reported internet ability did not predict use of the pedometer or website. Frequency of internet use, however, was associated with more frequent log-ons to the web-based intervention. For that reason, it may be difficult to engage participants who do not use the internet in web-based interventions. However, rates of internet use are increasing every year, and, consequently, acceptance and adoption of web-based interventions are also likely to increase [31].

A fraction of participants had difficulties using the pedometer (ie, obtaining step count feedback from the pedometer or uploading their step count data to the website). This barrier did not vary by age. Our results support that persons with COPD may benefit from pedometers that are simple to use and guidance on how to use them. While communication and education can influence acceptance and use, experience is also needed. Experimental research has shown that brief use of an eHealth application can decrease the expected difficulty or effort [32,33]. Offering participants more opportunity to become acquainted with the technology at baseline, or initial clinic visits, may increase their intention to use the device in the future.

### Strengths and Limitations

A major strength of this study is the use of the UTAUT conceptual framework to understand how age influences persons with COPD’s acceptance and use of a technology-mediated physical activity intervention. In addition, the participants in our sample were given an opportunity to incorporate the technology into their daily lives for 12 weeks, as opposed to some technology acceptance studies that explore participants’ initial impressions of technology and do not reflect actual use [34,35]. This was a secondary analysis using questions that were formed based on clinical interest and not originally based on the UTAUT. As such, the questionnaire did not use previously validated UTAUT questions. We used dichotomous survey questions to reduce participant burden and may have missed some granularity in participants’ opinions.

Although we used UTAUT to guide our secondary analysis, we were limited by the data that had been collected. The UTAUT model also includes the construct of social influence, which refers to the belief that others (eg, family, providers) believe that the individual should use the new technology [20]. This was not assessed as part of the original study. Along with age, UTAUT theorizes that gender, experience, and voluntariness influence technology adoption [21]. The current sample’s participants were mostly White male Veterans with COPD. Therefore, we are unable to examine if gender influences perceptions of ESC. These results might not be generalizable to others. Similarly, all patients had the same amount of experience with the intervention. Another limitation of studies of patient-facing technologies is the voluntary nature of the study; individuals who feel less comfortable with technology may be less likely to have enrolled in the study. Additionally, participants were asked to log on to the website at least once a week and wear the pedometer every day. Thus, these measures may also be indicative of compliance and not necessarily voluntary use of the technology. Future work would benefit from exploring these other UTAUT moderators (gender, experience, voluntariness) and age variations in technology acceptance among a more diverse sample of participants with COPD.

Another potential limitation is that these analyses include only the RCT participants randomized to the ESC intervention; there is a chance that type II error may have caused us to miss some age-related differences. A larger sample would statistically allow for more degrees of freedom within the model. However, among our sample, we were able to detect significant age-related differences for ease of finding time to engage in the intervention, belief that the step count goals were too high, and comfort reaching step goals. Therefore, we are confident we are powered to detect differences despite the small sample size.

### Conclusion

Veterans with COPD are likely to accept and use technology to promote physical activity. However, not all of them can easily adopt it. Those who use the internet more will be more inclined to use the web-based intervention. Thus, familiarizing participants with the various technologies may facilitate use of the technology. To encourage positive behavior change, interventions that use incremental goal setting should consider adapting automated goals by age so that participants can perceive them as attainable. If older adults can feel encouraged and confident, they will accept and use web-based interventions.

## Abbreviations

COPD: chronic obstructive pulmonary disease
ESC: Every Step Counts
FEV1: forced expiratory volume in the first second
FEV1% predicted: forced expiratory volume in the first second percent predicted
FVC: forced vital capacity
OR: odds ratio
RCT: randomized controlled trial
UTAUT: unified theory of acceptance and use of technology
